# Stochastic variational learning in recurrent spiking networks

**DOI:** 10.3389/fncom.2014.00038

**Published:** 2014-04-04

**Authors:** Danilo Jimenez Rezende, Wulfram Gerstner

**Affiliations:** ^1^Laboratory of Cognitive Neuroscience, School of Life Sciences, Brain Mind Institute, Ecole Polytechnique Federale de LausanneLausanne, Vaud, Switzerland; ^2^Laboratory of Computational Neuroscience, School of Computer and Communication Sciences, Ecole Polytechnique Federale de LausanneLausanne, Vaud, Switzerland

**Keywords:** neural networks, variational learning, spiking neurons, synapses, action potentials

## Abstract

The ability to learn and perform statistical inference with biologically plausible recurrent networks of spiking neurons is an important step toward understanding perception and reasoning. Here we derive and investigate a new learning rule for recurrent spiking networks with hidden neurons, combining principles from variational learning and reinforcement learning. Our network defines a generative model over spike train histories and the derived learning rule has the form of a local Spike Timing Dependent Plasticity rule modulated by global factors (neuromodulators) conveying information about “novelty” on a statistically rigorous ground. Simulations show that our model is able to learn both stationary and non-stationary patterns of spike trains. We also propose one experiment that could potentially be performed with animals in order to test the dynamics of the predicted novelty signal.

## 1. Introduction

Humans and animals are able to learn complex behavioral tasks and memorize events or temporally structured episodes. Most likely, learning and memory formation are intimately linked to changes in the synaptic connection strength between neurons. Long-term potentiation and depression of synapses can be induced by many different experimental protocols, and depend on voltage (Artola and Singer, 1993; Ngezahayo et al., 2000), spike-timing (Markram et al., 1997; Bi and Poo, 2001) as well as a subtle combination of timing, voltage, and frequency (Sjöström et al., 2001; Clopath et al., 2010). Spike-Timing Dependent Plasticity (STDP) has intrigued theoreticians, because it provides a local Hebbian learning rule for spiking neurons; local, here, means that the dynamics of the synapses is of the form $\frac{d}{dt}$*w_ij_* ∝ *h*(*post_i_, pre_j_*), where *pre_j_* is the set of pre-synaptic variables of neuron *j* (e.g., spike timing) and *post_i_* is the set of post-synaptic variables of neuron *i* (e.g., spike times and voltage) and *h* is an arbitrary functional.

Unsupervised learning through STDP has been repeatedly shown (Levy et al., 2001; Song and Abbott, 2001; Izhikevich et al., 2004; Morrison et al., 2007; Cateau et al., 2008; Gilson et al., 2009; Clopath et al., 2010) to yield connectivity structures that leads to non-trivial activity patterns in recurrent spiking networks.

With relation to neuroscience, unsupervised learning is most commonly related to developmental plasticity (Miller et al., 1989), formation of receptive fields (Song and Abbott, 2001) or cortical rewiring (Young et al., 2007). Indeed most early applications of unsupervised STDP concern the learning of feedforward connections and the formation of receptive fields (Gerstner et al., 1996; Kempter et al., 1999; Song et al., 2000; Song and Abbott, 2001). Unsupervised STDP will tune to the earliest spikes (Song and Abbott, 2001; Gerstner and Kistler, 2002; Guyonneau et al., 2005) and can perform Independent Component Analysis (Clopath et al., 2010; Savin et al., 2010).

On the level of behavioral neuroscience, human performance approaches in many psychophysical learning paradigms Bayes optimality, i.e., the best statistical model cannot perform better than humans do (Knill and Pouget, 2004; Körding and Wolpert, 2004). This supports the hypothesis that the brain is performing approximate inference, which implies that the brain has access to prior and posterior distribution of possible explanations of the observed data (Berkes et al., 2011).

These findings lead to the idea that the spiking activity of the brain constitutes a generative model, that is, a model of the joint distribution of percepts (observed spike trains induced by sensors) and hidden causes in the world (hidden spike train generated by neurons that are not directly affected by sensor spikes).

The ability to model hidden causes in the sensory data is important for both stationary and non-stationary situations. For stationary distributions of spike trains, hidden neurons are important to encode potential interpretations or explanations of spike patterns observed at the sensory neurons (visible neurons). For non-stationary sequences, hidden neurons are fundamental for representing the non-observed dynamics and to form long-term memories.

Various abstract Bayesian models have been proposed to account for this phenomenon (Körding and Wolpert, 2004; Deneve, 2008; Nessler et al., 2009). However, it remains an open question whether optimization in abstract Bayesian models can be translated into plausible learning rules for spiking neurons. If one considers only stationary input patterns, an explicit relation between Bayesian inference and synaptic plasticity has been suggested (Habenschuss et al., 2012). Moreover, it has been suggested recently (Pecevski et al., 2011; Shao, 2012) that spiking networks with biologically plausible dynamics can produce stationary samples from Deep Boltzmann Machines (Salakhutdinov and Hinton, 2009) and more general Bayesian networks. In the following we drop the limitations of stationary spatial patterns and consider a recurrent network of stochastically spiking neurons as a generative model of spatio-*temporal* spiking patterns.

Learning the synapses between hidden neurons in recurrent models has been recognized as a difficult problem, as these synapses only contribute indirectly to the activity of the visible neurons and as recurrence leads to the “vanishing gradient” problem (Bengio et al., 1994). Conversely, in machine learning the algorithms known to be efficient for learning graphical and recurrent models are typically non-local (Jaakkola and Jordan, 2000; Bhatnagar et al., 2007; Sutskever et al., 2008; Salakhutdinov and Hinton, 2009). That is, in order to efficiently perform parameter updates in these models, their learning rules either take into account the entire state of the model or it requires that information to propagate in a non-causal maner through the synapses.

Therefore, the locality constraints imposed by biology constitutes one of the major challenges in transforming those algorithms into biologically plausible learning rules.

Even in biology, however, certain types of non-local signals participate in the learning process, notably through neuromodulators which can convey information about the global state of the network or external information (e.g., reward or surprise) (Izhikevich, 2007; Schultz, 2008; Fremaux et al., 2010).

Here, we derive a principled learning rule for unsupervised learning in recurrent spiking networks that relies only on quantities that are locally available at the synapse: pre-synaptic activity, post-synaptic activity and a global modulatory signal.

The key innovation of our model compared to earlier studies (Brea et al., 2011; Jimenez Rezende et al., 2011) lies in the computation of a global modulating signal which is a linear superposition of local terms and can therefore be interpreted as the diffusion of a neuromodulator in the extra-cellular medium.

Furthermore our global neuromodulating signal conveys information about *novelty* or *surprise* on statistically rigorous grounds, providing interesting links with findings relating surprise and plasticity (Gu, 2002; Ranganath and Rainer, 2003; Yu and Dayan, 2005).

We show with simulations based on synthetic data that our proposed learning mechanism is capable to capture complex hidden causes behind the observed spiking patterns and is able to replicate, in its spontaneous activity, the statistics of the observed spike trains.

Finally, we provide an application of our model to a hypothetical novelty detection task where a simulated agent (e.g., a rat) is inserted into a maze with specific properties (views, rooms, topology of the maze). Our model, simulating the “brain” of this agent, successfully captures the statistical properties of this environment. We show that, after learning, our model is capable of distinguishing the original environment from another environment that differs only in its topology (relative location of the rooms). Additionally, we predict the “expected dynamics” of a neuromodulator signaling “novelty” as the agent traverses the virtual maze. We propose that this hypothetical experiment could be developed into a real experiment.

## 2. Materials and methods

### 2.1. Neuron model

The neuron model used in our simulations is a generalized linear model (GLM) in the form of a Spike Response Model (SRM) with escape noise (Gerstner and Kistler, 2002; Jolivet et al., 2006). The spike train of a neuron *j* for times *t* > 0 is denoted as $X_{j}(t)=\sum_{t_{j}^{f}\;\in \;\left\{t_{j}^{1},\ldots ,t_{j}^{N_{s}}\right\}}\delta \left(t-t_{j}^{f}\right)$, where {*t*^1^_*j*_, …, *t^N_s_^_j_*} is the set of spike timings.

We model the membrane potential of a neuron *i* as

(1) $$u_{i}(t)=\sum_{j}w_{ij}\phi_{j}(t)+\eta_{i}(t),$$

where $\eta_{j}(t)=-\eta_{0}\int_{0}^{t}dse^{-\frac{\left(t\;-\;s\right)}{\tau_{\text{adapt}}}}X_{j}(s)$ is the adaptation potential, *w_ij_* is the synaptic strength between neurons *i* and *j* and ϕ*_j_*(*t*) is the potential evoked by an incoming spike from neuron *j*. The evoked potentials are modeled by a simple exponential filter $\phi_{j}(t)=\int_{0}^{t}dse^{-\frac{\left(t\;-\;s\right)}{\tau }}X_{j}(s)$ implemented as a differential equation

(2) $${\dot{\phi }}_{j}(t)=\frac{1}{\tau }(X_{j}(t)-\phi_{j}(t)),$$

where τ is the time constant of the membrane potential.

The spikes are generated by a conditioned Poisson process with exponential escape rate (Jolivet et al., 2006). That is, the conditional instantaneous firing intensity ρ_*i*_(*t*) is taken to be

(3) $$\rho_{i}(t)=\rho_{0}\exp\left[\frac{u_{i}(t)-\vartheta }{\Delta u}\right],$$

where ϑ and Δ*u* are physical constants of the neuron. However, we will keep ρ*_i_*(*t*) as an arbitrary function of *u_i_*(*t*), ρ_*i*_(*t*) = *g*(*u_i_*(*t*)), in all our derivations and we specify the exponential form (Equation 3) only when performing the simulations (see further below). Equations (1–3) capture the simplified dynamics of a spiking neuron with stochastic spike firing.

**Figure 1:**
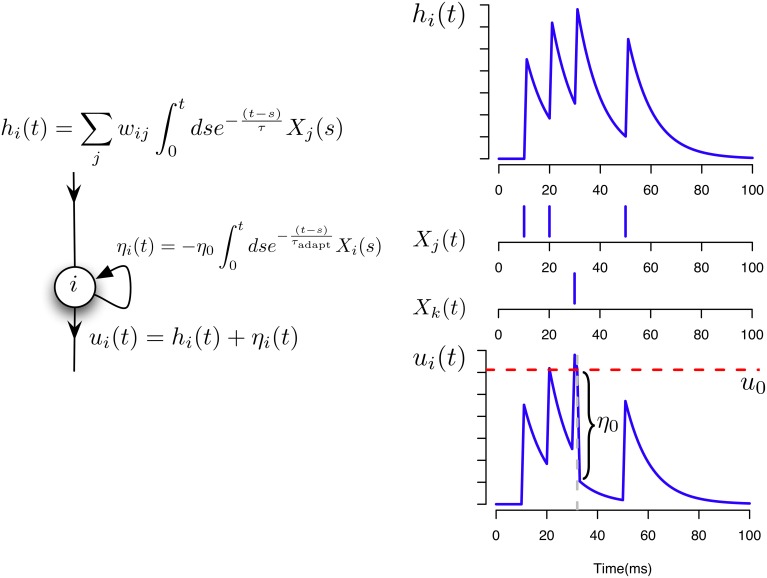
**Neuron and synaptic models**. Illustration of the different contributions to the total membrane potential of a neuron *i* in our model.

In the following simulations we assume that two different neurons *i* and *j* can have at most two common synapses, *w_ij_* and *w*_*ji*_. The neuron model and the potentials contributing to its activation are illustrated in Figure 1.

In the following sections, we will first introduce the theoretical framework in which we derive our learning rule. The learning mechanism is then derived in several steps, followed by simulations showing that our model and learning rules are capable of capturing complex spatio-temporal features in the input spike trains, reproduce them in its spontaneous activity and perform statistical inference on the hidden causes of provided data.

### 2.2. A principled framework for learning

**Figure 2:**
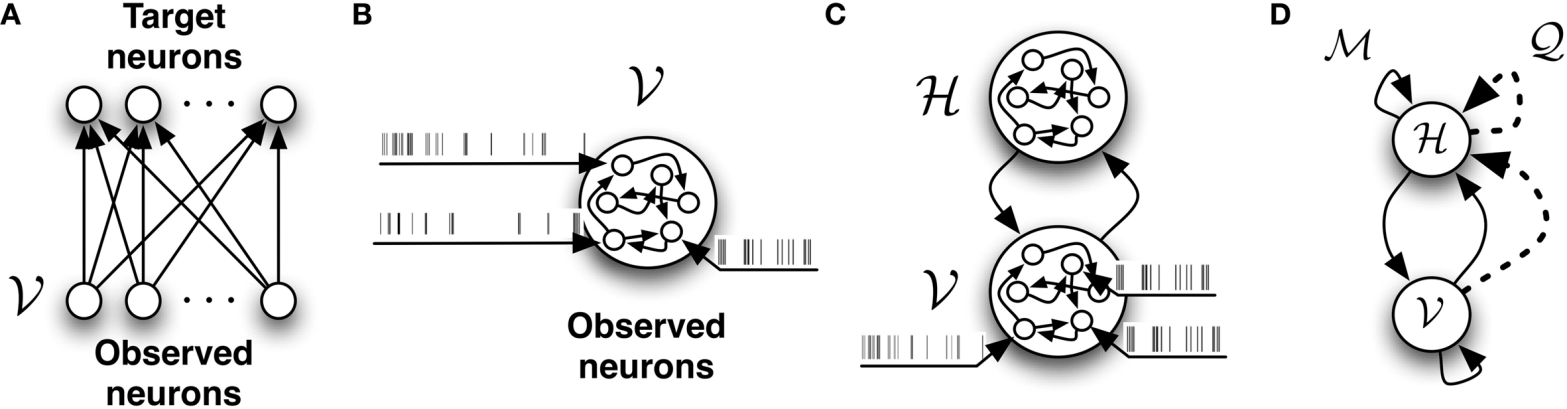
**The different network architectures discussed in this study. (A)** Feed-forward, fully observed spiking network. It defines a conditional model of the target spiking patterns given the input patterns. **(B)** Fully connected and fully observed spiking network. It defines a generative model of the observed data. **(C)** Network decomposed in two pools of neurons: neurons which receive data or observed neurons 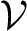 and neurons which do not receive data or hidden neurons 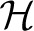. Both pools of neurons are fully connected. **(D)** The network with 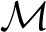-synapses (solid links) and 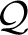-synapses (dashed links) which provides the infrastructure required for learning the generative model.

In the following we consider a stochastic, fully connected network 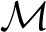 composed of two sets of neurons which are functionally distinct. The first group which we call *observed* or *visible* neurons (denoted by 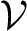) represents an ensemble of neurons which receive data in the form of spike trains. The spike trains of the visible neurons will be referred to as *X*_
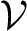
_. These neurons are exposed to the external world. The second set, which we call *hidden* neurons (denoted by 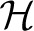) does not directly receive data from the external world. The spike trains of the hidden neurons will be referred to as *X*_
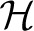
_. Their role is to provide “compressed explanations” for the observed data. The topology of this network is illustrated in Figure 2C. In the absence of external drive, the spontaneous activity of the hidden neurons will contribute to the firing of the observed neurons. The spike trains of the entire network comprising both observed and hidden neurons will be indicated simply as *X*.

Our network defined in this way constitutes a generative model of spike trains with hidden neurons. In the following we interpret synaptic potentiation and depression as a form of optimization of this generative model. More precisely, we assume that synaptic plasticity (the “learning rule”) is trying to increase the likelihood of the observed spike trains under the model.

In what follows, we review the calculation of the complete-data log-likelihood log p(*X*_
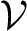
_, *X*_
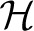
_) for a recurrent network of point-process neurons.

From Equations (1–3) it follows that the probability *P_i_*(*t^f^_i_* ∈ [*t, t* + Δ*t*]|*X*(0…*t*)) of producing a spike in the infinitesimal interval [*t, t* + Δ*t*] by the *i*th neuron conditioned on the past activity of the entire network *X*(0…*t*) and provided that ρ*_i_(t)* Δ*t* « 1 is given by

(4) $$P_{i}\left(t_{i}^{f}\in [t,t+\Delta t]|X(0\ldots t)\right)\;\approx \;\rho_{i}(t)\Delta t,$$

and the probability *P_i_*(*t^f^_i_* ∉ [*t*, Δ*t*]|*X*(0…*t*)) of producing no spike in the same interval is given by

(5) $$P_{i}\left(t_{i}^{f}\notin [t,t+\Delta t]|X(0\ldots t)\right)\;\approx \;(1-\Delta t\rho_{i}(t)).$$

Therefore, by discretizing a finite time interval [0, *T*] into *N* sufficiently small bins [*t_k_*, *t_k_* + Δ*t*] with *k* = 1 … *N* so that there is at most one spike in each bin and assuming independence between neurons within the infinitesimal bins we can write the probability *P*(*X*(0…*T*)) of producing a spike train *X_i_(t)* = ∑*_t^f^_i__*δ(*t* − *t^f^_i_*) for all neurons in the network as

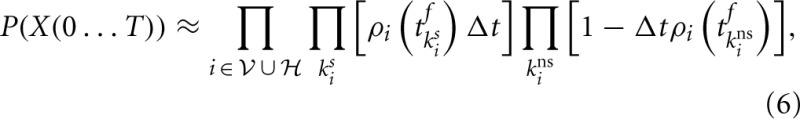



where *k_i_*^*s*^ and *k_i_*^*ns*^ labels the bins with one spike and bins without spike from neuron *i*, respectively. Taking the limit *N* → ∞ of Equation (6) divided by the volume Δ*t^K^*, where *K* is the total number of spikes in the interval [0, *T*] we obtain the probability density

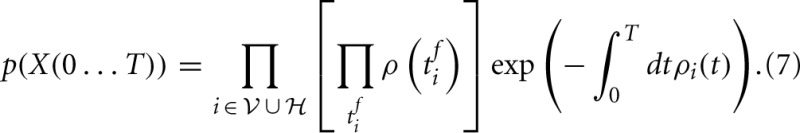



The log-likelihood corresponding to Equation (7) can be compactly written as

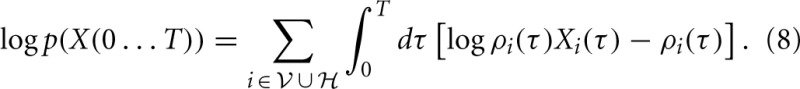



It should be stressed that the log-likelihood (Equation 8) is not a sum of independent terms, since the instantaneous firing rate of each neuron depends on the entire past activity of all the other neurons through Equations (1–3).

In the following sections we derive a plasticity rule that will attempt to maximize the log-likelihood of the observed data log *p*(*X*_
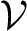
_). For this, we briefly review the calculation of the gradient of the observation likelihood in absence of hidden neurons and then we introduce our method for approximating its gradient when there are hidden neurons.

### 2.3. Fully observed network

The gradient of the log-likelihood of fully observed networks of SRM neurons and similar point-processes have been studied in detail in Paninski (2004) and Pfister et al. (2006). In what follows we review the calculation of the gradients of the data log-likelihood (Equation 8) with respect to the synaptic weights and then discuss a simulation revealing the limitations of the model.

In a network without hidden neurons as illustrated in Figures 2A,B, the data log-likelihood (Equation 8) reduces to a simple form

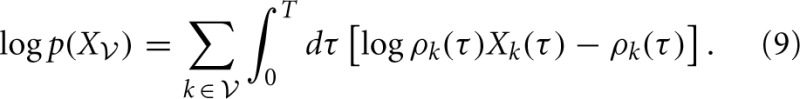



In Paninski (2004) and Pillow et al. (2004) it is shown that the optimization problem defined by the log-likelihood (Equation 9) is convex. Therefore its global maximum can be found by gradient ascent.

The gradient of Equation (9) with respect to the synaptic efficacies *w_ij_* is given by

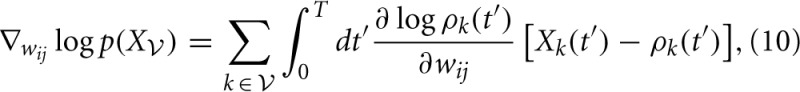



where the gradients $\frac{\partial \log\rho_{k}(t^{\prime })}{\partial w_{ij}}$ are obtained by differentiating the firing rate function (Equation 3):

(11) $$\frac{\partial \log\rho_{k}\left(t^{\prime }\right)}{\partial w_{ij}}=\delta_{ki}\frac{g^{\prime }\left(u_{k}\left(t^{\prime }\right)\right)}{g\left(u_{k}\left(t^{\prime }\right)\right)}\phi_{j}\left(t^{\prime }\right)$$

We conclude that the gradient ∇*_w_ij__* log *p*(*X*_
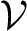
_) can be calculated in a purely local manner. More precisely, an update of the weights according to gradient ascent Δ*w_ij_* ∝ ∇*_w_ij__* log *p*(*X*_
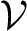
_) yields a learning rule that is simply a “trace” of a product of two factors: A first factor ϕ_*j*_(*t*′) that depends only on the presynaptic activity and a second factor $\frac{g^{\prime }\left(u_{k}\left(t^{\prime }\right)\right)}{g\left(u_{k}\left(t^{\prime }\right)\right)}\left[X_{k}\left(t^{\prime }\right)-\rho_{k}\left(t^{\prime }\right)\right]$ that depends on the state of the postsynaptic neuron. Moreover, the gradient (Equation 10) has been shown to yield a simplified form of STDP (Pfister et al., 2006). The notion of “trace” (i.e., a temporal average of some quantity) will return for other learning rules later in this paper.

**Figure 3:**
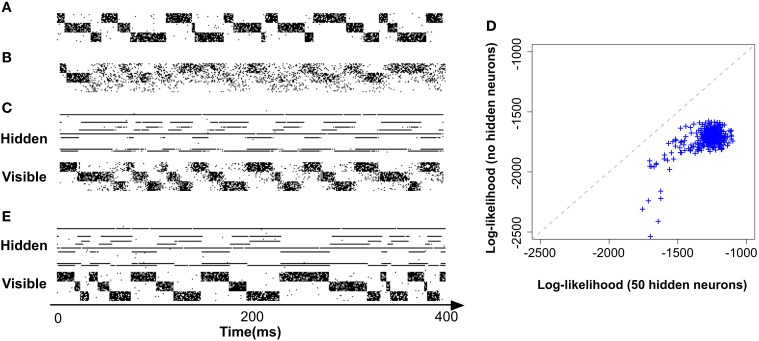
**Models with hidden neurons succeed where a fully observed model fails**. Here we compare our model (online with variance reduction, see Materials and Methods) with and without hidden neurons. **(A)** Sample from the training data. **(B)** Sample activity from the visible neurons of a learned model without hidden neurons. From the generated samples we can see that a model with only visible neurons can capture some correlations present in the data (note the formation of “downward” moving blurred patterns) but fails at capturing the long-term structure of the pattern. **(C)** Sample from a learned model with 50 hidden neurons: hidden neurons' spikes (top) and visible neurons' spikes (bottom). Note that the learned hidden representation unambiguously represents the global state of the visible neurons, generating different spiking patterns for each distinc phase of the training pattern. **(D)** Relative performance of the models with (vertical axis) and without (horizontal axis) hidden neurons measured by their data log-likelihood. The blue points indicate the relative evolution of the data log-likelihood of both models during a learning session. The model with latent neurons has systematically a better performance (higher log-likelihood) than the model without hidden neurons. **(E)** Model with hidden neurons in inference mode. The activity of the hidden neurons from the 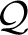-network (top) forms a higher-level representation of the observed spike trains (bottom).

In order to expose the weakness of the fully observed model described above, we test its performance on a task which consists of learning “stair patterns” involving 3 groups of 10 visible neurons, which are probabilistically activated using low (1 Hz) and high (700 Hz) firing rates. The activations generate a sequential pattern where each group remains active for a duration drawn from a Gaussian distribution with mean 30 ms and standard deviation 10 ms (truncated at positive values), (Figure 3A). This benchmark is interesting firstly because it requires the formation of memories on the order of three times the membrane time constants of the single neuron dynamics and secondly because it requires the ability to learn the appropriated transition probabilities. Our simulations show that a fully observed network is not capable of learning such patterns, as can be seen by looking at the samples produced from the learned network (Figure 3B). However, a model with 50 hidden neurons (with the learning rule discussed further below) can learn the distribution (Figures 3C,D).

Moreover, even if the fully observed network could learn the data distribution it would not provide any useful representation of the data, while a network with hidden neuron naturally forms higher-level representations of the incoming data (Figure 3E)(top). For precise details concerning the numerical simulations and evaluations, see further below.

This simulation corroborates the intuition that a network consisting of visible neurons only is rather limited in scope. We therefore turn in the following to a more general network consisting of both visible and hidden neurons.

### 2.4. Partially observed network

In the following we first introduce our model that includes hidden neurons. We derive our learning rule and introduce a few modifications to improve its performance. Finally, we show with simulations that the resulting model can learn spiking patterns that couldn't be learned by a model without hidden neurons.

In a model that includes hidden neurons (that is, neurons not directly connected to the incoming data), the marginalized likelihood of the visible neurons is obtained by integrating over all possible hidden spike trains *X*_
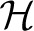
_,

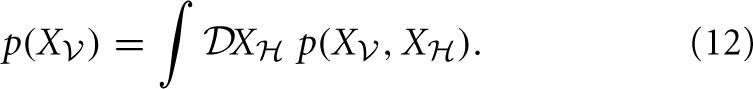



In the following we introduce the variational approximation scheme for approximating Equation (12). The variational approach consists of approximating a complex distribution *p* by a simpler distribution *q* and provides a flexible generalization of the expectation-maximization (EM) algorithm (see Jaakkola and Jordan, 2000; Beal and Ghahramani, 2006).

We are interested in approximating the posterior *p*(*X*_
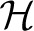
_|*X*_
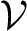
_) by another distribution over spike trains *q*(*X*_
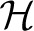
_|*X*_
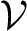
_). We optimize the parameters of the distribution *q*(*X*_
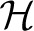
_|*X*_
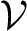
_) by minimizing the KL-divergence

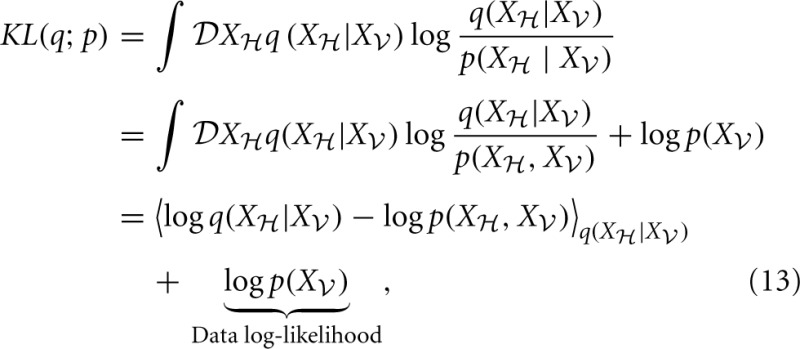



where 〈*f*(*X*)〉_*p*_ = ∫
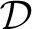
*Xf(x)p(x)*. The first term in Equation (13) is known in statistical physics as the *Helmholtz free energy* (Landau and Lifshitz, 1980),

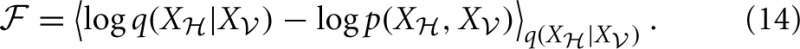



The second term in Equation (13) is simply the data log-likelihood. Since the KL-divergence *KL*(*q*; *p*) between two distributions *q* and *p* is non-negative (Gibbs and Su, 2002), the free energy (Equation 14) is an upper bound on the negative log-likelihood. Therefore, we can redefine the problem of maximizing the data log-likelihood log *p*(*X*_
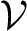
_) with respect to the parameters of the generative model *p* as the double optimization problem of minimizing the free energy 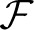 with respect to the parameters of *q and* with respect to the parameters of the original model *p*.

The attractiveness of such an approach for deriving biologically plausible rules comes from the fact that the distribution *q* is arbitrary (as long as it has the same support as the true distribution). Therefore we can either choose it in order to simplify the calculations or to improve the model's compliance with known biological constraints, such as causality and locality of the weight updates. These interesting properties of the variational approximation have been explored by a diversity of models (Dayan, 2000; Friston and Stephan, 2007; Jimenez Rezende et al., 2011; Brea et al., 2013; Nessler et al., 2013).

In what follows, we postulate that the posterior distribution of the hidden spike trains of the network 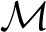 can be well approximated by another recurrent network of spiking neurons which we call the network 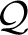. This network is composed of the same neurons as the original model, except that it has different synaptic connections. Its connectivity is depicted in Figure 2D.

The assumption of an “inference” network 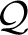 is analogous to the *recognition model* introduced in Dayan (2000) for the Helmholtz machine.

Our model differs from the Helmholtz machine, as introduced in Dayan (2000), in two key aspects: (1) The Helmohtz machine is a model for stationary data, i.e., it cannot readily model temporal sequences; (2) Although the recognition network is introduced in a variational framework, the proposed learning rule is not attempting to minimize a free energy (there are different cost functions for the generative and recognition models) whereas our learning rule is explicitly attempting to minimize the free energy associated to the model.

In other words, our model consists of a *single* set of neurons with two sets of synaptic weights that can be turned on and off independently (possibly through the action of specific neuromodulators). The first set of weights, which we will refer to as *w*^
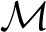
^_*ij*_, parameterizes the original generative model 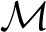. The second set of weights, which we will refer to as *w*^
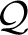
^_*ij*_ parameterizes the network 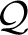. The topology of the network 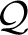 is restricted and excludes connections toward the visible neurons from hidden and other visible neurons.

In the following, all the quantities (e.g., membrane potentials and firing rates) that are computed using the parameters *w*^
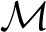
^_*ij*_ (i.e., with 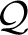 turned off) will have the superscript 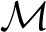. Analogously, all the quantities that are computed using the parameters *w*^
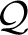
^_*ij*_ (i.e., with 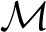 turned off) have the superscript 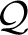.

When driven solely by the observed spike trains and by the weights *w_ij_*^
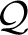
^, the activity of the hidden neurons *X*_
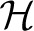
_ is, by construction, an approximated sample from true the posterior distribution *p*(*X*_
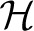
_|*X*_
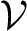
_) provided by the distribution *q*(*X*_
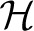
_|*X*_
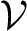
_). Therefore, if we want our model to perform approximated Bayesian inference on the most likely hidden causes of an observed spike train *X*_
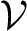
_ we just have to run it with the synapses *w_ij_*^
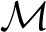
^ turned off. Conversely if we want to produce a sample from the learned generative model, we have to run it with the synapses *w_ij_*^
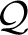
^ turned off.

In the next sections we derive stochastic estimators of the gradients of the free energy 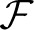 with respect to the synaptic weights *w_ij_*^
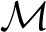
^ and *w_ij_*^
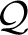
^ in a biologically plausible manner.

We show that the naive gradients obtained for *w_ij_*^
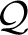
^ are problematic since their variance grows quadratically with the size of the network. We then introduce a simple modification to reduce the variance of the obtained gradients based on techniques from reinforcement learning. Finally we modify the gradient estimators so that they turn into “on-line” parameter updates.

### 2.5. Stochastic gradients

The complete data log-likelihood 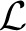
^
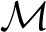
^ of the model 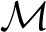 and 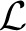
^
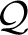
^ of the model 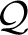 are given by

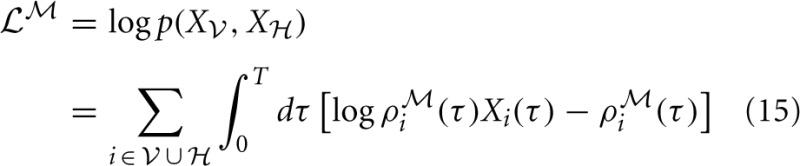



and

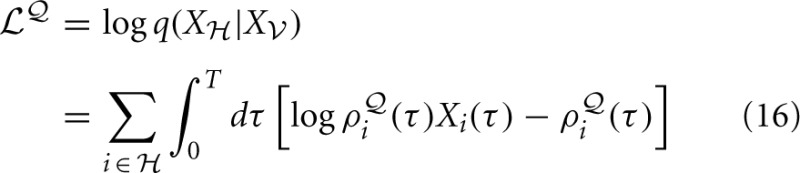



respectively.

The free energy (Equation 14) corresponding to the log-likelihoods (Equations 15, 16) is given by

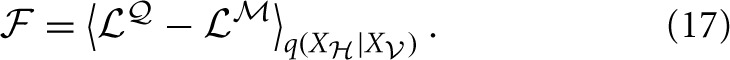



In the following we simplify the notation and write 〈•〉_*q*_ instead of 〈•〉_*q*(*X*_
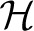
_|*X*_
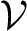
_)_. We wish to write the learning equations for both *w*^
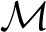
^_*ij*_ and *w*^
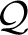
^_*ij*_ as simple gradient descent on the free energy:

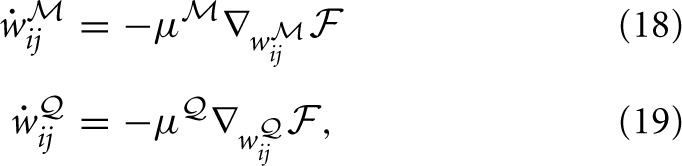



where μ^
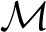
^ and μ^
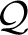
^ are learning rates for the networks 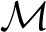 and 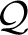, respectively. The exact gradients ∇_*w*^
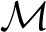
^_*ij*__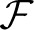 and ∇_*w*^
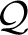
^_*ij*__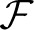 are difficult to evaluate analytically since we cannot compute the required expectations. Therefore we resort to unbiased stochastic approximations of those gradients.

The calculation of the gradient ∇_*w*^
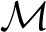
^_*ij*__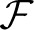 is analogous to the fully observed case and is given by

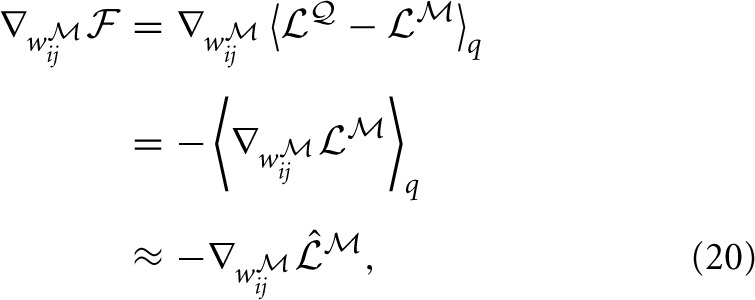



where 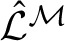 is a point-estimate of the complete data log-likelihood of our generative model obtained by computing the required traces from a single simulation of the network 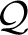 in the interval [0, *T*]. Similarly, the stochastic gradient with respect to *w*^
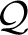
^_*ij*_ is given by

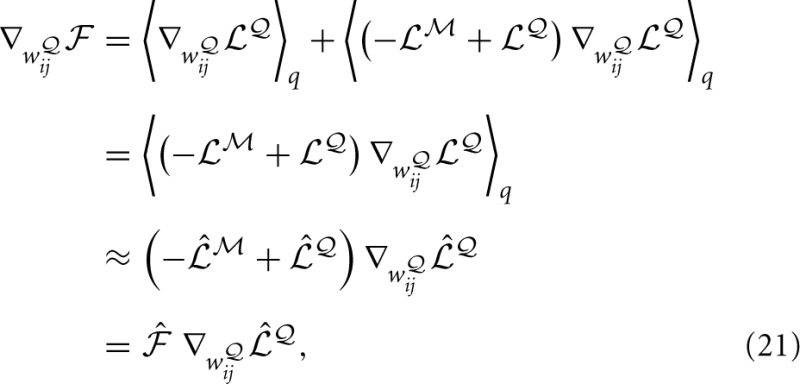



where we have used the fact that 
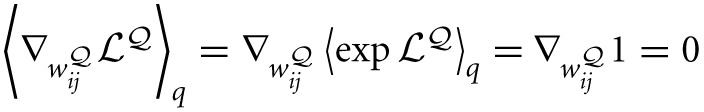
 and 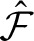 is the point-estimate of the free energy,

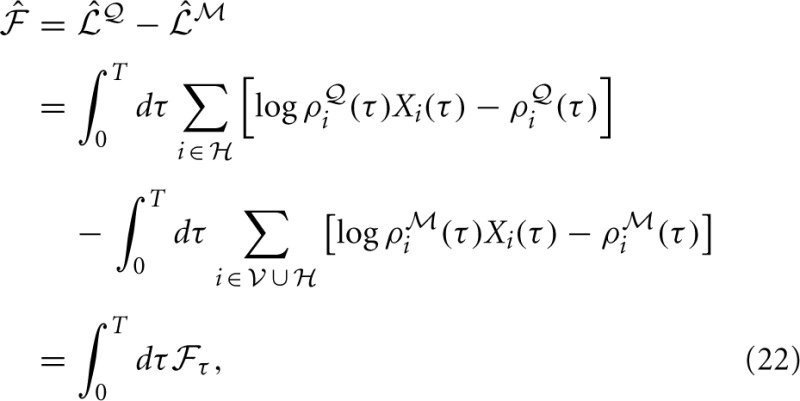



where we have defined the “instantaneous free energy” 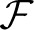
_τ_ as

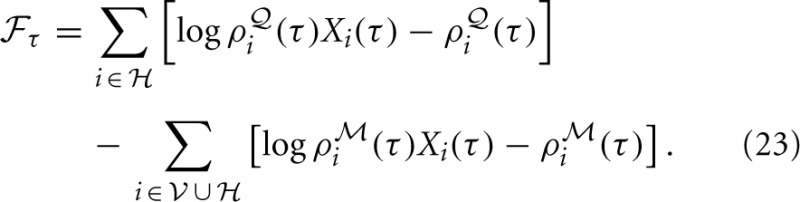



Note that during learning the activity of the visible neurons is driven purely by the observed spike trains while the activity of the hidden neurons and related quantities (e.g., ρ^
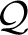
^_*i*_ and ρ^
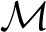
^_*i*_) is driven by the 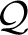-network. Expanding the remaining gradients in Equations (20, 21) using the chain rule we obtain the “batch mode” learning equations

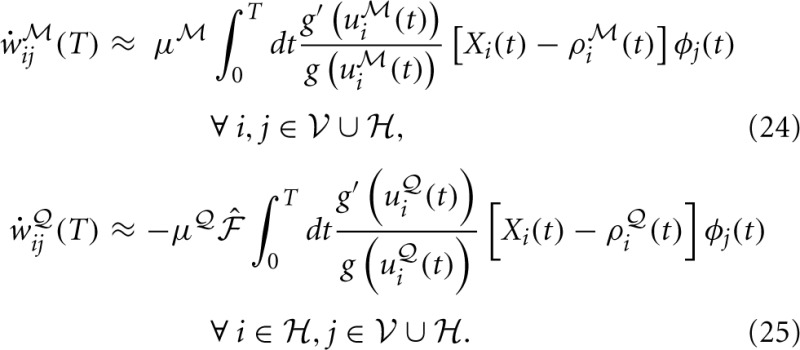



Note that the learning rule (Equation 24) is the same as in the fully observed network case in Equation (10). The learning rule (Equation 25) for the network 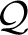 is similar, but contains an additional modulation factor, the point estimate of the free energy 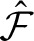, which appears as a global signal that modulates the learning of the network 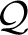. Since 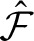 provides a lower bound on the data log-likelihood, the free energy measures how much the recent history of observed spike trains “fits” the generative model defined by the network 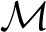. The assumption of a globally available signal conveying information about *reward* or *surprise* is standard in the reinforcement learning literature.

The naive stochastic gradients (Equations 24, 25) are not efficient in practice. Even though they constitute unbiased estimators of the true gradients, their *variance is prohibitively high*. We address this problem below.

### 2.6. Reducing the variance of the gradients

Stochastic gradients of the form Equation (25) have been extensively studied in the reinforcement learning literature and several approaches with different levels of complexity have been proposed for reducing their variance (Munos, 2005; Bhatnagar et al., 2007). In the following, we sketch some theoretical arguments for why gradients of the form of Equation (25) should be expected to scale badly with the size of the network.

Let *N* be the number of neurons in the network and *T* be the time window on which we compute the relevant quantities. Equation (25) contains the free energy 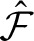 which is, according to Equation (22), a sum of traces (integrals on the interval [0, *T*]) for each neuron. Therefore, in a weak-coupling scenario, the estimator (Equation 25) is the integral on the interval [0, *T*] of a sum of *N* weakly correlated terms in the free energy, that we assume to have some typical variance σ^2^_0_ and mean *m*. Under these assumptions, the variance of the gradient (Equation 25) scales approximately as

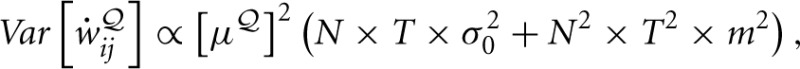



That is, as the size of the network grows, the variance of the gradient (Equation 25) grows with the square of the number of neurons. A naive solution would be to decrease the learning rate as μ^
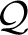
^ ∝ 1/*N* but this would make learning too slow for larger networks.

In the following, we adopt a simple baseline removal approach to reduce the variance of our gradient estimator. That is, we simply subtract the mean 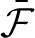 of the free energy 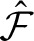, calculated as a moving average across several previous batches of length *T*, from the current value 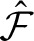
(*T*). This yields the learning rule

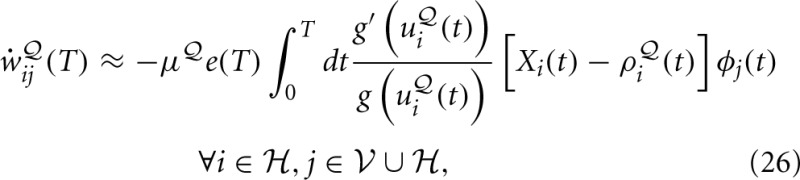



where we have introduced the “free energy error signal” *e*(*T*) = 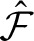
(*T*) − 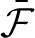.

With this simple change, we can see that the variance of our gradient scales as

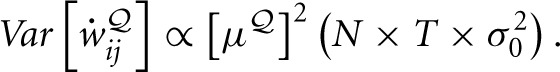



The baseline removal trick is not a solution to the problem but it drastically reduces the variance of our estimator when the size of the network is increased.

Note that the quadratic or linear growth of the gradient's variance with the number of neurons is not just an artifact of our variational approximation. The arguments presented in this section apply to any learning rule that has the form of local Hebbian traces modulated by global signals that are composed of several weakly correlated terms. For instance, the learning rule proposed in Brea et al. (2011) also falls into this category.

### 2.7. Online vs. batch learning

The gradients that define our learning scheme (Equations 24, 26) are given in terms of quantities accumulated over a given time interval [0, *T*]. In this sense, we have derived a “batch” learning rule and we would have to wait until the end of the interval [0, *T*] in order to apply the changes in the parameters of our model.

However, compatibility with biology requires that we have an online version of our algorithm. This can be approximately achieved if, instead of accumulating the traces required for calculating the gradients on time interval [0, *T*] and applying the parameters updates in the end, we replace the traces by moving averages and apply the parameter updates at *every* time step. The modified learning rules can be formulated as follows. At each synapse, we have “Hebbian traces” *H*^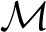, 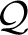
^_*ij*_(*t*) that keep track of pre- and post-synaptic activity and evolve according to

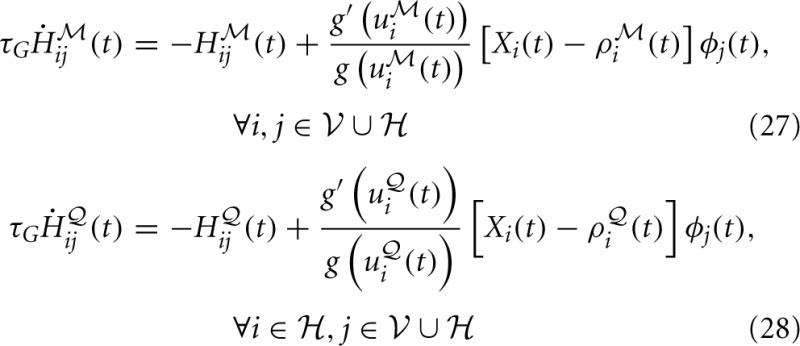



where we have introduced a time constant τ_*G*_ controlling the time-scale of the moving averages. Similarly, the online estimate of the error signal *e_N_(T)* is obtained by replacing time integrals in the interval [0, *T*] with moving averages

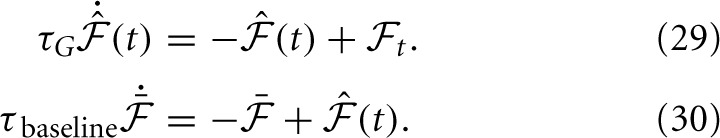



That is, 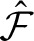 is a “short term” moving average of the instantaneous free energy 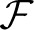
_*t*_ (Equation 23) with time-scale τ_*G*_ while 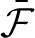 is a “long term” moving average of 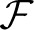
_*t*_ with a longer time-scale depending on τ_*G*_ and τ_baseline_. Note that the “error signal” *e_N_(T)* can also be interpreted as an instantaneous surprise measure relative to the slow “background” surprise level 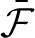. The updates of the weights uses the Hebbian traces and fixed learning rates μ^
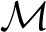
^ and μ^
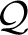
^

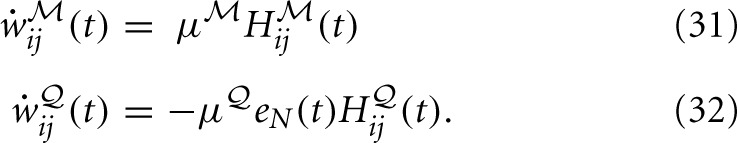



**Figure 4:**
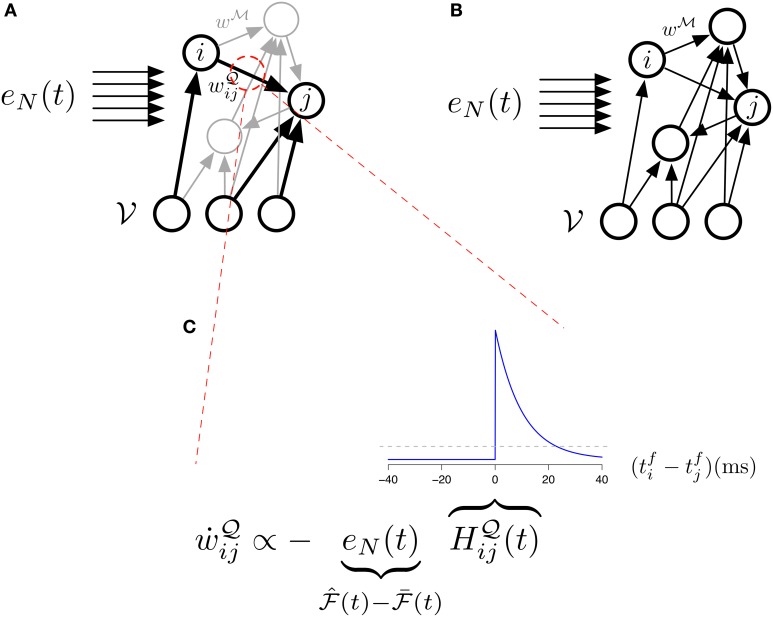
**The different studied architectures and three-factor STDP. (A)** Illustration of our main model (Equations 31, 32) with its two sets of synapses 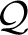 (black) and 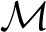 (gray). The “error signal” *e_N_(t)* modulates the learning of the 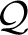-synapses. **(B)** Illustration of the simplified model (Equations 33, 34) with its single set of synapses 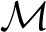. The “error signal” *e_N_(t)* modulates the learning of the 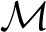-synapses. **(C)** Learning in the main model's 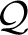-synapses is triggered by the covariance between the Hebbian trace *H*^
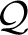
^_*ij*_ and the moving average of free energy 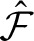. The inset blue curve indicates the shape of the Hebbian trace term as a function of the time interval between pre and post synaptic spikes. The dotted gray line corresponds to *H*^
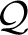
^_*ij*_ = 0 (no weight change). A positive covariance will induce synaptic depression (left quadrant of blue curve) while a negative covariance will induce synaptic potentiation (right quadrant of blue curve). A null covariance would induce a centered random walk on the synaptic weights.

Thus the update of the 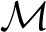-network is given by a “Hebbian” rule whereas the update of the 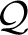-network follows a three-factor rule with the surprise as a global factor. This architecture is illustrated in Figure 4A and the three-factor rule for the 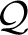-synapses is illustrated in Figure 4C. Another way of interpreting the learning rule (Equation 32) is that it is simply proportional to the covariance between the Hebbian trace *H*^
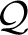
^_*ij*_ and the moving average of the free energy 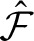. If they are uncorrelated, the expected change in the parameters will be zero and the synaptic weights will just perform a centered random walk.

### 2.8. A simplified model

Since the variational distribution *q* in Equation (13) can be arbitrary, one could imagine a model simpler than the one derived in the previous sections which consists in approximating the posterior distribution of the hidden neurons directly by the forward dynamics of the generative model. In practice this approximation amounts to constraining the synaptic weights *w*^
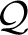
^_*ij*_ of the 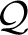-network to be equal to the synaptic weights *w*^
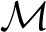
^_*ij*_ of the 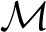-network for *i* ∈ 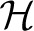 and *j* ∈ 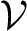 ∪ 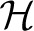.

Under this constraint, the learning equations (31) and (32) reduces to

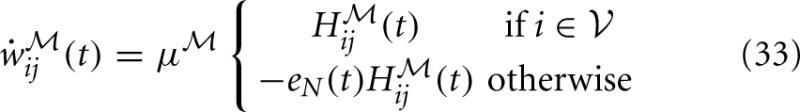



and the instantaneous free energy simplifies to a sum over the observed neurons only

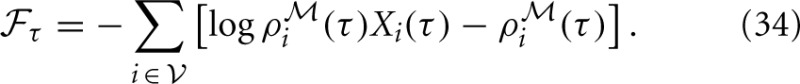



The architecture of this model is illustrated in Figure 4B The idea of using the forward-dynamics as a proposal distribution for the posterior has been used in Brea et al. (2011) and Brea et al. (2013), where the proposals are then weighted by an importance-sampling scheme to better represent the true posterior distribution over the hidden activity.

Further below (see results) we show that, at least in the context of the variational learning discussed here, this approximation does not outperform our more general model.

### 2.9. Numerical simulations

All the simulations in this study are based on a discrete-time version of the Equations (31, 32). The spiking process is approximated by taking 1 − exp[−*dtρ(t)*] as the probability of producing one spike in the finite time bin [*t*, *t* + *dt*]. The values of the parameters used in this study are reported in Table 1.

**Table 1:** **Parameters used in the simulations**.

**Parameter**	**Description**	**Value**
*dt*	Time discretization interval	1 ms
τ	Membrane potential time constant	10 ms
η_0_	Adaptation potential strength	0.1 mV
τ_adapt_	Adaptation potential time-scale	10 ms
ρ_0_	Firing rate scale	1 kHz
ϑ	Firing threshold	0 mV
Δ*u*	Firing sensitivity to the membrane potential	1 mV
μ^ 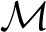 ^	Learning rate for the model network 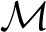	0.00001
μ^ 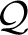 ^	Learning rate for the recognition network 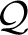	0.00001
τ_*G*_	Time scale for the moving averages of the gradients and free energy	10 ms
τ_baseline_	Time scale for the moving average of mean free energy	100 ms

The initial synaptic weight of both 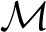 and 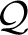 were sampled from a Gaussian distribution with mean zero and standard deviation of 0.01.

For all experiments, the training data consists of binary arrays with ones indicating spikes and zeros indicating no-spike in the corresponding time bin. The training data-sets are organized in batches of 200 ms which are sequentially presented to the model. During our training sessions, each learning epoch corresponds to 500 presentations of data batches. In other words, each epoch corresponds to a total of 100 s of spiking data. During the learning phase, the visible neurons are exactly driven by the data spike trains, that is, they are forced to spike or to not spike in the exact same way as the data samples at each time bin. During the spontaneous activity phase the networks are running without any external drive.

The log-likelihood of test data was estimated by an importance sampling procedure. Given a generative model with density *p*(*x_v_, x_h_*) over observed variables *x_v_* and hidden variables *x_h_*, importance sampling allow us to estimate the density *p*(*x_v_*) of a data point *x_v_* as

(35) $$p(x_{v})={\langle p(x_{v}|x_{h})\rangle }_{p(x_{h})}={\langle p(x_{v}|x_{h})w(x_{h},x_{v})\rangle }_{q(x_{h})},$$

where *q*(*x_h_*|*x_v_*) is an arbitrary distribution with same support as *p*(*x_h_*) and *w*(*x_h_, x_v_*) = *p*(*x_h_*)/*q*(*x_h_*|*x_v_*) is the importance weight. The Equation (35) can be rewritten in terms of the point-estimated of the free energy (Equation 22) as follows

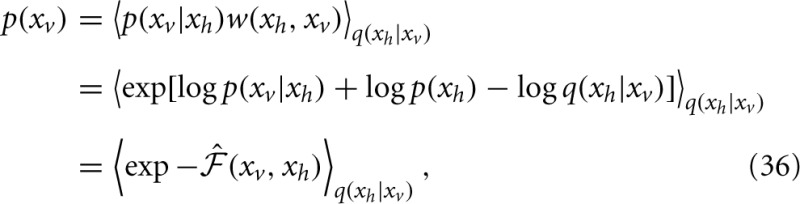



where 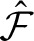
(*x_v_, x_h_*) is a point-estimator of the free energy.

Using Equation (36) we estimate the log-likelihood of a observed spike-train by sampling several times from the network 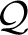 and taking the average of the exponentiated point-estimator of the free energy (Equation 22). For our applications, we have generated 500 samples of duration 100 ms from the network 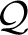 per estimation.

## 3. Results

The learning rules derived above (see Materials and Methods) come in four different variants. First, a batch-based naive gradient descent rule. Second, a variant that reduces the variance of the gradient estimator. Third an online version of the variance-reduced rule that is biologically more plausible than a batch rule. Finally, a simplified version of the model where the 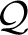-network is merged with the 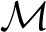-network in a specific manner.

### 3.1. Variance reduced rule vs. naive rule

**Figure 5:**
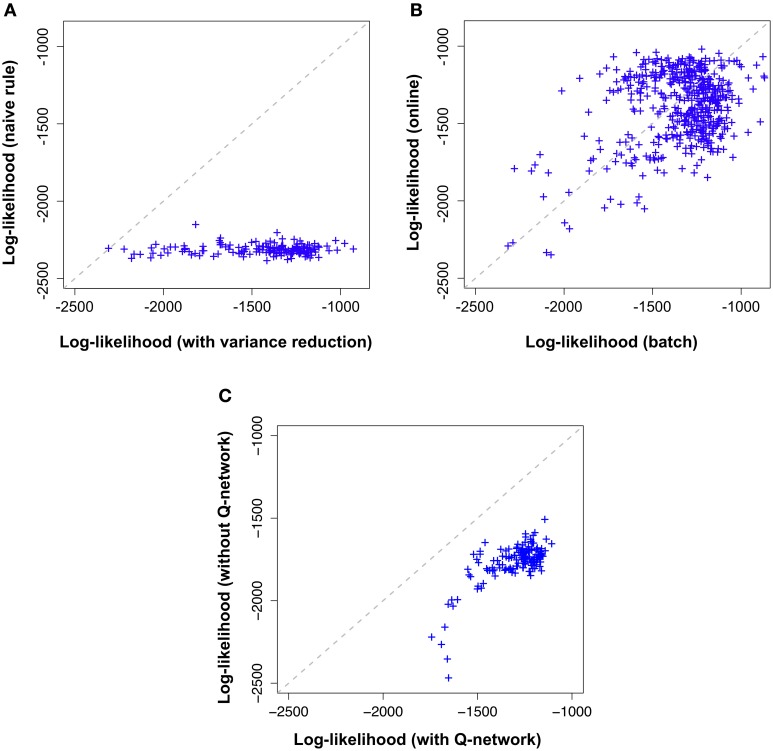
**Variance reduction and** 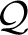
**-network are important while on-line approximation is not**. Comparison of the different flavors of the proposed model across 100,000 learning epochs on the “stairs pattern” task. Shown log-likelihoods where estimated by importance sampling every 500 epochs during learning. Likelihood values (crosses) further to the right (high log-likelihood) correspond to the end of learning while the cross on the diagonal marks the beginning of learning (epoch 1). **(A)** Model with variance reduction (horizontal axis) achieves a higher log-likelihood than a naive model without variance reduction (vertical axis). **(B)** Batch model with variance reduction(horizontal axis) and on-line model with variance reduction (vertical axis) exhibit a similar evolution of log-likelihoods. **(C)** Online-model with variance reduction (horizontal axis) performs better than the simplified model (without the 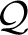-network) with variance reduction defined by Equations (33, 34) (vertical axis).

Naive gradient descent on the free energy yields the batch learning rule (Equation 25). Tested on the stairs-patterns (Figure 3A) using a network of 30 visible neurons and 30 hidden neurons generates very slow learning. In Figure 5A we show that the learning rule with the variance reduction (Equation 26) performs substantially better than the naive gradient (Equation 25). Both networks have the same number of visible and hidden neurons, but the first network is trained with the gradient given in Equation (25) whereas the second network is trained with the gradient defined in Equation (26). The data log-likelihood of both networks is approximated by importance sampling after every learning epoch.

In Figure 5A, along the horizontal axis we plot, across several epochs the log-likelihood of the model using the learning rule with variance reduction and along the vertical axis the log-likelihood of the model without variance reduction (i.e., the naive batch gradient rule). We find that the log-likelihood of the variance reduced model is consistently above the log-likelihood of the naive model, indicating an increase in learning speed of more than a factor 100.

### 3.2. Online algorithm vs. variance reduced batch algorithm

Online algorithms are biologically more plausible than batch algorithms which require storage of intermediate results. Here we show that the online learning rules (Equations 31, 32) induce only minor impairments of the performance compared to the batch rules (Equations 24, 26).

Both versions of the model were trained on the same “stair pattern” task and the results are shown in Figure 5B. Along the horizontal axis we indicate the log-likelihood of the batch model and along the vertical axis that of the online model, across several epochs of learning. Both performances are strongly correlated, indicating that the online approximation does not introduce any major impairment in the model for this particular dataset.

### 3.3. Forward dynamics vs. the 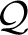-network

Here we show that the simplified model defined by the Equations (33, 34) does not reach the performance of the more general model defined by Equations (31, 32).

Both versions of the model were trained on the same “stair pattern” task and the results are shown in Figure 5C. Along the horizontal axis we indicate the log-likelihood of our model with 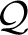-network and along the vertical axis that of the simplified model, across several epochs of learning. Both performances are correlated. However, the simplified model has clearly lower log-likelihoods than the complete model. This result suggests that the forward dynamics of the generative model may provide a poor approximation to the true posterior distribution of the hidden neurons compared to having an independently parameterized inference network.

### 3.4. Hidden representations and inference

To show that our model is not only learning a prior that represents the data but can also form interesting hidden representations and perform inference on the hidden explanations of the incoming data, we use the 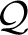-network of a model with 50 hidden neurons, trained on the stairs dataset Figure 3A. The activity of the hidden neurons of the model during sampling (or “dreaming”) are shown on Figure 3C (top). As we can see just by visually inspecting the activity of the hidden neurons, they form an unambiguous representation of the activity of the visible neurons in this simple example. Conversely, by running the model on “inference mode” (i.e., with the synapses *w*^
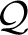
^ activated) can also see that the model is capable of performing inference on the causes of the incoming data Figure 3E.

### 3.5. The role of the novelty signal

The resulting online rule given in Equation (32) for is a Hebbian-type plasticity rule modulated by a global novelty signal *e_N_(t)* where *e_N_(t)* is a measure of surprise relative to a slow moving average of the free energy. We repeat the learning rule for the 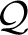-network from Equation (32),

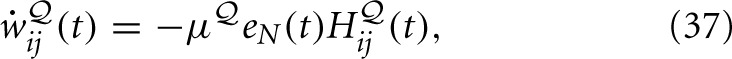



where *H*^
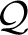
^_*ij*_(*t*) is a synaptic trace that keeps track of “Hebbian” coincidence between pre- and post-synaptic activity.

The Hebbian trace

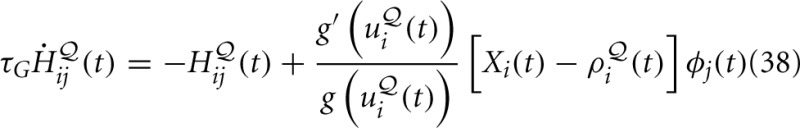



is activated by the product of a voltage-dependent post-synaptic factor 
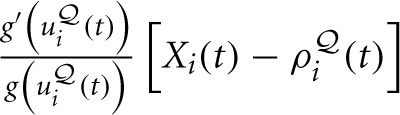
 and a presynaptic EPSP caused by presynaptic spike arrival.

It is known that the brain is able to detect novelty and to broadcast novelty related signal across large brain regions (Gu, 2002; Ranganath and Rainer, 2003). In order to illustrate the dynamics of the novelty signal in our model we describe a task which could be transformed into a real animal experiment.

We place an agent (e.g., a rat) in a maze and let it explore it. During the exploration, the agent will learn the topology of the maze through a combination of visual and proprioceptive information. If the agent is suddenly transported to another maze with many similar but a few different parts we expect this change to trigger some novelty signal in the brain of the agent whenever it encounters the parts of the new maze that differ from the learned maze. In order for the agent's brain to detect a change in the environment, it must first learn a sufficiently accurate model of the environment. We hypothesized that our recurrent neuronal network learns the structure of the environment and at the same time provides an online surprise signal when it encounters a “novel” situation. Such a novelty signal could be compared to recordings of different neuromodulators.

**Figure 6:**
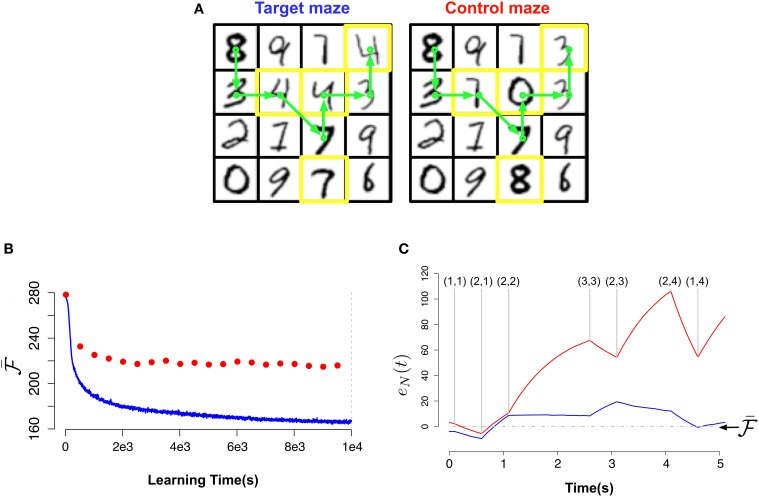
**Novelty-gated Hebbian learning. (A)** The target maze (left) and test maze (right). Each one of the 16 rooms in the maze is represented by a random MNIST digit. Transitions are only possible between neighbor rooms. The test maze was generated from the target one by randomly replacing a few rooms (indicated with yellow borders). A sample trajectory is shown with the green arrows. **(B)** Evolution of the slow moving average of the free energy 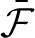 as a function of the amount of observed data for the target maze (blue) and the mean free energy of the same model when “teleported” to the control maze (red) every 500 s. **(C)** Fine-grained temporal details of the fast free energy traces *e_N_(t)* while traversing the sample trajectory for the target maze (blue) and for the test maze (red). Visited rooms are indicated by the annotated coordinates at each change-point. During the intervals between two consecutive change-points the agent remains in the same room.

We created two virtual mazes composed of 16 “rooms” arranged as a square lattice where only neighboring rooms are accessible from each other: a first one which the agent will learn, the target maze, indicated in Figure 6A (left) and the test maze Figure 6A (right) which is similar to the first but with a few, randomly chosen rooms, replaced as indicated in Figure 6A by the yellow squares. Note that the only way to detect the difference between the two mazes in this simulation is to actually learn the exact connectivity graph of the rooms, because the rooms are themselves identical in both mazes.

The views corresponding to each room were generated by randomly choosing images of handwritten digits from the MNIST dataset (LeCun and Cortes, 2010). The MNIST images are 28×28 gray-scale images of handwritten digits. We converted the pixel-values to firing rates in the range [0.01 Hz, 9 Hz]. To keep the simulations simple, time was considered in abstract units for these simulations (with time steps of 100 ms instead of 1 ms).

For the “brain” of the agent we used a recurrent binary network with 30 hidden neurons and 28×28 visible neurons. In order to train this network, data batches where produced by recoding the activity of the visible neurons while the agent performs random trajectories of 100 time-steps in the target maze. Each learning epoch corresponds to 500 presentations of these data-batches to our model.

In Figure 6B we plot the “slow” moving average of the free energy 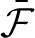 as a function of the learning time during the exploration phase for the target maze (blue curve) and for the test maze (red dots). The reported free energy for the control maze was measured every 5000 s for a path of length 50 s. As we can see, in the beginning of the learning the model is unable to distinguish between both mazes (both have high free energy). But as the model learns the target maze, it successfully identifies the test maze as “unfamiliar” (attributing low free energy to the target maze and much higher free energy to the test maze).

In Figure 6C we plot for both mazes the free energy error signal *e_N_(t)* for the sample trajectory shown in Figure 6A. From Figure 6C (blue) we can see that *e_N_(t)* fluctuates around zero for the learned maze but deviates largely from zero for the test maze.

This result suggests that if animals have a neurophysiological correlate of the “free energy error signal” *e*(*T*) introduced in Equation (26) we should look for activity bursts when the animals traverses unexpected situations (e.g., when traversing the test maze from position (2,1) to position (2,2)).

Moreover we should expect a substantial increase in the variance of the changes in synaptic weights when moving from a learned maze to an unfamiliar maze due to the change in the baseline of the surprise levels.

## 4. Discussion

We have proposed an alternative to the learning algorithms previously proposed in Brea et al. (2011) and Jimenez Rezende et al. (2011) for learning a generative model of spike trains defined by recurrent spiking networks. Our new model combines techniques from variational learning and reinforcement learning to derive a new efficient synaptic plasticity rule.

The resulting (see Materials and Methods) online rule for synapses is a Hebbian-type trace modulated by a global novelty signal. The Hebbian terms are traces of products of pre-synaptic terms (EPSPs) and post-synaptic terms. Similar gradients have been studied in Pfister et al. (2006) where they are found to yield STDP-like dynamics. Importantly, the global modulating signal, Equation (23) is a *linear superposition* of terms locally computed by each neuron so we can interpret it as the diffusion of a neuromodulator in the extra-cellular medium.

The original feature of the proposed model is that it uses an auxiliary recurrent spiking network in order to approximate the posterior distribution of the hidden spiking activity given the observed spike trains in an on-line manner. Using this auxiliary network allowed us to derive a learning rule which is based on local gradients modulated by slow non-local factors conveying information about “novelty.”

We have shown that naive stochastic gradients derived in such a framework are not viable in practice due to their high variance (which may grow quadratically with the number of hidden neurons). Deriving viable learning rules thus requires finding low-variance and unbiased estimators of the gradients defined in Equations (24, 25). In this paper we have only reduced this problem as our learning rule (Equation 26) still has a variance that grows (linearly) with the number of hidden neurons.

Our proposed learning algorithm, has potential applications for finding functional networks from recorded neurophysiological data. Since it can learn a recurrent spiking network that approximatively “explains” the data. Taking into account external currents injected into the network would be straightforward.

We also provide an on-line neural estimator of novelty/surprise and make experimentally testable predictions about its dynamics. The estimator is a quantity that naturally emerges from the statistical principles behind our framework instead of being an *ad hoc* quantity.

From the biological literature, it is known that novelty or surprise is, at least partly, encoded in neuromodulators such as ACh (Ranganath and Rainer, 2003; Yu and Dayan, 2005). Moreover, neuromodulators are known to affect synaptic plasticity (Gu, 2002). We suggested a hypothetical animal experiment that could test predictions concerning a novelty signal and its potential relation to plasticity.

## 5. Related work

The framework in which we derive our model is very general and has been used in different ways in previous works (Dayan, 2000; Friston and Stephan, 2007; Jimenez Rezende et al., 2011; Brea et al., 2013; Nessler et al., 2013). The uniqueness of the present works relies on the combination of methods from variational learning and reinforcement learning yielding a learning rule that is both biologically plausible and efficient.

The main difference between our model and models that exploit more analytical properties of the variational approximation (explicitly computing expectations and covariances terms in the free energy) (Friston and Stephan, 2007; Jimenez Rezende et al., 2011) is that, although these analytical approximations may provide gradient estimators with much lower variance and typically yield more scalable algorithms, these methods are intrinsically non-local and further approximations are required in order to obtain a biologically plausible learning rule.

An interesting learning algorithm for recurrent spiking networks which approximates the gradients of the KL-divergence (Equation 13) using an importance sampling technique has been proposed in Brea et al. (2011) and Brea et al. (2013). Their algorithm does not use an auxiliary network to approximate the activity of the hidden dynamics conditioned on the observed spike trains. Instead, the generative forward dynamics of the model is used as a proposal distribution which is then weighted by an importance-sampling approximation.

Another important family of algorithms proposed in Habenschuss et al. (2012) and Nessler et al. (2013), heavily relies on approximating recurrent neural networks with soft winer-takes-all (WTA) dynamics. One advantage of assuming a WTA dynamics is that the computation of the gradients of the KL-divergence (Equation 13) greatly simplifies, yielding a simple local learning rule. A limitation of their approach is that the model does not take into account the full temporal dynamics during inference.

## Funding

Financial support was provided by the Swiss National Science Foundation (SystemsX) as well as by the European Research Council (Grant Agreement no. 268 689), and the European Community's Seventh Framework Program (Grant Agreement no. 269921, BrainScaleS).

### Conflict of interest statement

The authors declare that the research was conducted in the absence of any commercial or financial relationships that could be construed as a potential conflict of interest.
